# Eccentricity dependent auditory enhancement of visual stimulus detection but not discrimination

**DOI:** 10.3389/fnint.2013.00052

**Published:** 2013-07-19

**Authors:** Stephanie Gleiss, Christoph Kayser

**Affiliations:** ^1^Max Planck Institute for Biological CyberneticsTübingen, Germany; ^2^Institute of Neuroscience and Psychology, University of GlasgowGlasgow, UK

**Keywords:** multisensory, perception, cross-modal facilitation, visual field, audio–visual integration

## Abstract

Sensory perception is enhanced by the complementary information provided by our different sensory modalities and even apparently task irrelevant stimuli in one modality can facilitate performance in another. While perception in general comprises both, the detection of sensory objects as well as their discrimination and recognition, most studies on audio–visual interactions have focused on either of these aspects. However, previous evidence, neuroanatomical projections between early sensory cortices and computational mechanisms suggest that sounds might differentially affect visual detection and discrimination and differentially at central and peripheral retinal locations. We performed an experiment to directly test this by probing the enhancement of visual detection and discrimination by auxiliary sounds at different visual eccentricities and within the same subjects. Specifically, we quantified the enhancement provided by sounds that reduce the overall uncertainty about the visual stimulus beyond basic multisensory co-stimulation. This revealed a general trend for stronger enhancement at peripheral locations in both tasks, but a statistically significant effect only for detection and only at peripheral locations. Overall this suggests that there are topographic differences in the auditory facilitation of basic visual processes and that these may differentially affect basic aspects of visual recognition.

## INTRODUCTION

Combining the information provided by the different sensory modalities strongly influences perception and our interaction with the environment. For example, many studies have explored the conditions under which sounds can facilitate the performance in visual psychophysical tasks. In general such audio–visual interactions can reflect the perceptual integration (i.e., the merging) of feature related information provided by each modality, or they can reflect the general facilitation of perception by additional (i.e., auxiliary) stimuli that do not carry directly task relevant information. In principle both these interactions can entail faster reactions in multisensory environments (Posner et al., 1976; Gielen et al., 1983), the more precise estimate of sensory variables (Alais and Burr, 2004b; Ernst and Bulthoff, 2004), or the facilitation of sensory detection (Alais and Burr, 2004a; Lippert et al., 2007; Chen et al., 2011). In some conditions, this can even result in an illusory multisensory percept (McGurk and MacDonald, 1976; Sekuler et al., 1997; Shams et al., 2000). While the perceptual conditions for audio–visual interactions have been explored in good detail, important specific questions about their functional properties remain unresolved (Chen et al., 2011; Spence, 2011, 2013).

Sensory perception comprises the detection of salient events in space and time as well as their discrimination with regard to specific features or configural properties and recognition. Multisensory studies have often focused on either of these aspects of perception or glossed over these differences. However, differentiating stimulus detection and discrimination may be of particular relevance to the auditory facilitation of basic visual processes. In particular so as this kind of multisensory interaction likely involves early visual cortices or specific visual pathways (Foxe et al., 2000; Foxe and Schroeder, 2005; Ghazanfar and Schroeder, 2006), as suggested not only by psychophysical studies (Jaekl and Harris, 2009; Jaekl and Soto-Faraco, 2010) but also by functional imaging work (Giard and Peronnet, 1999; Molholm et al., 2002; Watkins et al., 2006; Noesselt et al., 2010; Werner and Noppeney, 2010). In addition, neuroanatomical studies have shown that auditory cortices project to primary and secondary visual areas and revealed that these projections are comparatively stronger in the peripheral than the central visual field (Falchier et al., 2002, 2010; Rockland and Ojima, 2003). Together with initial reports about a variable sensitivity of central and peripheral visual stimuli to multisensory influences (Shams et al., 2002; Tremblay et al., 2007), this may point to a functional specificity of an auditory enhancement with regard to retinal eccentricity. Given that sensory detection and discrimination are generally considered to rely on peripheral and central visual fields, respectively, any eccentricity dependence of auditory influences may also result in a differential impact on stimulus detection and discrimination.

Yet, very few studies have addressed the specificity of multisensory interactions with regard to retinal position or task nature using the same stimulus context, general task procedure, or within the same subjects. Those who did, found somewhat contradicting results, which suggested either a functional specificity with respect to eccentricity (Leo et al., 2008), or absence of such (Fiebelkorn et al., 2011), and similarly found diverging results for a differential impact on stimulus detection or discrimination (Andersen and Mamassian, 2008; Jaekl and Harris, 2009; Chen et al., 2011). This highlights that a potential benefit of acoustic stimuli for visual perception could not only vary between tasks but could also be dependent on the general experimental procedure, calling for a more systematic comparison of how sounds can enhance either perceptual function across retinal locations.

We here tested whether auxiliary sounds specifically enhance visual detection or discrimination performance at central or peripheral locations within the same subjects and relative to basic multisensory co-stimulation. Note that “detection” here refers to the differentiation of spatial stimulus attributes, while we use discrimination to refer to the differentiation of other configural properties. To this end we used a task that allowed testing both the detection and discrimination of visual stimuli while manipulating both the informativeness of an additional sound and the target eccentricity. We then contrasted multisensory performance in the presence of two types of sounds, one that provided no information about any feature involved in the task other than stimulus timing (i.e., basic multisensory co-stimulation), and one that provided no direct information about the visual task but reduced uncertainty within the space of all possible visual stimuli. Importantly, we contrasted two conditions including sound, rather than contrasting one condition with and one without sound, to avoid confounding benefits arising from basic auditory co-stimulation. Such effects of multisensory co-stimulation have been convincingly described in many previous studies and are not the focus here (see e.g., Lippert et al., 2007; Jaekl and Soto-Faraco, 2010; Chen et al., 2011; Spence, 2011). We found that in this context only visual detection was enhanced by the presence of an informative sound and only at peripheral locations, despite the overall performance being comparable for both locations and tasks.

## MATERIALS AND METHODS

### GENERAL PROCEDURES

Adult volunteer subjects (aged 19–32, both sexes) were paid to participate in the experiments. All reported normal hearing, normal or corrected to normal vision and gave written informed consent prior to participation. The experiments were approved by the joint ethics committee of the University Clinic and the Max Planck Institute for Biological Cybernetics Tübingen and were conducted according to the Declaration of Helsinki. Experiments were performed in a sound-attenuated and dark room. Visual stimuli were presented on a gamma-corrected monitor (24′′, 60 Hz) positioned 57 cm from the subject’s head, while acoustic stimuli were presented binaurally using a Sennheiser In-Ear headphone (Model PMX 80). Stimulus presentation was controlled from Matlab (Mathworks) using routines from the Psychophysics toolbox (Pelli, 1997). Sound levels were calibrated using a condenser microphone (Bruel&Kjær 4188) and a sound level meter (2238 Mediator; Bruel&Kjær). The subjects head was stabilized using a chin-rest and a computer keyboard was used to collect their responses.

**FIGURE 1:**
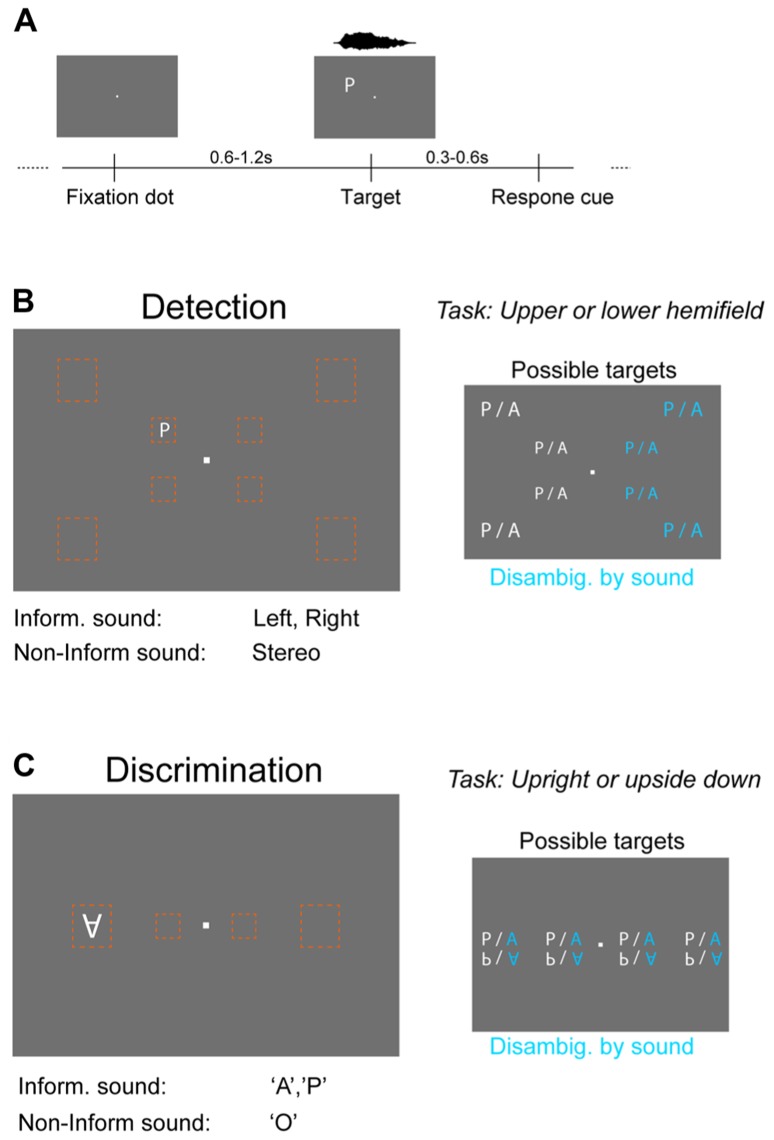
**Behavioral paradigms and stimuli.(A)** Both the detection and discrimination task were based on the same general procedure. Visual targets were shown for one frame (~16 ms) at a random time point after the onset of a fixation dot. Subsequently a question mark cued subjects to respond by pressing a button on a keyboard. Targets were always accompanied by a sound, which could be either “informative” or “non-informative.” **(B)** Detection task. Visual targets (letters “A” or “P”) were presented at eight different locations (orange boxes) of the visual field that were either central (5° from fixation dot) or peripheral (14°). Subjects had to indicate in which hemifield relative to the horizontal midline the target appeared. The informative sound reduced spatial uncertainty about target location by being lateralized (left or right speaker only), while the non-informative sound provided only temporal co-stimulation (stereo sound). The reduction in stimulus uncertainty by the informative sound is indicated in blue on the right. **(C)** Discrimination task. The same visual targets were presented at four positions along the horizontal midline at either central (5°) or peripheral (14°) locations. Subjects had to indicate the orientation of the letter (upright, upside down). The informative sound reduced uncertainty about the letter (sound “A” or ”P”; stereo), while the non-informative sound (“O”) provided only temporal co-stimulation.

Subjects (*n* = 13) performed both a detection task and a discrimination task, the order of which was randomized across subjects. Both tasks (see **Figure 1A**) were based on a two-alternative forced choice (2-AFC) procedure and involved the detection/discrimination of dim visual targets presented at different positions on a neutral gray screen (3 cd/m^2^ background luminance). Targets were shown for one frame (~16 ms) in a random period (600–1200 ms, uniform) after the onset of a central fixation dot. After a delay period (300–600 ms) a question mark appeared centrally on the screen cueing the subject to respond by pressing a button. Subjects were instructed to respond “as accurately as possible.” Visual targets were always accompanied by a sound whose onset was synchronized to the onset of the visual target and which was presented at an average intensity [root mean square (RMS)] of 65 db(A) sound pressure level (SPL). Synchronization was verified using a photodiode and oscilloscope. For each task trials were grouped in blocks of 256 trials and each subject performed four blocks per task. In between blocks subjects made a break of 5–15 min duration (self-paced).

### AUDIO–VISUAL TASKS

Both tasks used letters as visual stimuli (“A,” “P”) and the sounds of these two plus a third letter (“O”) spoken by a female English speaker. Visual letters (termed targets) were presented at different locations of the visual field relative to the horizontal midline, at different sizes and orientations and at two intensities levels (brightness) as described in the following. In each task targets could appear either at central visual locations (5° from the fixation dot) or at peripheral locations (14°) and the size of the visual target was scaled with the eccentricity to achieve similar levels of performance. Specifically, targets were scaled in proportion to the decrease in spatial visual acuity with eccentricity (Anstis, 1974). Acuity measurements show that targets at 14° should be about 2.8 times larger than at 5° to account for decreasing visual acuity. We hence scaled letters to be 1.3° high at 5° and to be 3.6° high at 14° eccentricities. This resulted in very similar performance levels for central and peripheral targets. In each task there were 16 different target configurations (positions, letters, or orientations) and each was presented at two intensities that were chosen to yield about 70% correct for the darker and 80% for the brighter value. These values were chosen for each subject using a separate testing procedure measuring psychometric curves (without sounds, using only one letter). The different target configurations, intensities, and sound conditions (see below) were randomized independently for each subject individually. The sounds did not provide direct evidence about the variable of interest for the primary visual task. However, they could either provide evidence to reduce uncertainty within the space of all possible visual targets (termed “informative sound”), or they were not informative about any of these variables and provided basic multisensory co-stimulation (termed “non-informative sound”). The letters “A” and “P” were used, rather than for example the acoustically more similar pair “A” and “U,” for the following reason. Preliminary tests had revealed that letters of similar shapes (e.g., A vs. U) make the discrimination task considerably more difficult and introduce an imbalance in overall performance levels between tasks. The use of letters that are more different in shape allowed us to balance performance across tasks at fixed visual intensities, an important factor for the present study.

#### Detection task

This task required subjects to report in which horizontal hemifield the visual target appeared (above or below the horizontal midline) and to indicate this by pressing a corresponding (“upper” or “lower” arrow) button on the keyboard (**Figure 1B**). Targets (“A” or “P”) could appear at eight different positions (four above and four below the horizontal midline; four to the left and four to the right of the fixation dot) that were either central (at 4° horizontal and 3° vertical distance from fixation dot) or peripheral (11.5° horizontal and 8° vertical distance). Sounds were either informative by being co-lateralized (left or right) with the visual target (sound on only one ear) or uninformative (stereo-sound). Both sounds were scaled to have comparable perceptual loudness, with lateralized sounds having an RMS intensity of 65 dB(A) SPL. The stereo sound was adjusted to have same perceived loudness as the mono sounds using an average rating of three independent observers. In the experiment a fixed relative scaling was used for all participants. For a given visual intensity the informative sound reduced the uncertainty about the target from 16 configurations (2 letters × 8 positions) to 8 configurations (2 letters × 4 positions) of which the letter was irrelevant for the task, hence leaving four positions that needed to be evaluated to detect the target. These four positions consisted of two eccentricities (analyzed separately) and two positions relative to the fixation dot (above or below). This position was the task relevant dimension that needed to be identified.

#### Discrimination task

This task required subjects to report the orientation of the visual target (upright or upside down) and to indicate this by pressing a corresponding (“upper” or “lower” arrow) button on the keyboard (**Figure 1C**). Targets (“A” or “P”) could appear at four different positions along the horizontal midline (two left and two right-hand of the fixation dot) that were either central (5° eccentricity) or peripheral (14°). Sounds were either informative about the letter currently presented by matching this (sound “A” and “P,” respectively) or were uninformative by reflecting a neutral letter (sound “O”). Sounds were presented stereo and had same RMS intensity. For a given visual intensity the informative sound reduced the uncertainty about the target from 16 configurations (2 letters × 2 orientations × 4 positions) to 8 configurations (2 orientations × 4 positions). These four positions consisted of two eccentricities (analyzed separately) and two positions relative to the fixation dot (left or right) that were irrelevant to the discrimination task. The orientation was the task relevant dimension that needed to be identified.

For each task we analyzed performance separately for each visual intensity and eccentricity and pooled over the task irrelevant dimension (letters or position). The main contrast of interest was the difference between informative and non-informative sounds. We verified that subjects did not exhibit a bias toward either response in the detection (percentage of responses using button 1 and 2 were 49.0 ± 1.4% vs. 51.0%) and discrimination tasks (48.4 ± 1.8% vs. 51.6%). We also directly verified that performance levels were similar across visual eccentricities and tasks (see Results).

## RESULTS

We tested subjects in visual detection and discrimination tasks in which visual targets were accompanied by sounds that either reduced the overall uncertainty within stimulus space or which did not provide any such information and simply constituted multisensory co-stimulation. By contrasting these two multisensory conditions we tested for multisensory enhancements specifically attributable to informative auxiliary sounds, without probing for direct multisensory integration of feature-specific information provided by two modalities, i.e., the sound did not provide direct information about the primary variable relevant for the visual task (Alais and Burr, 2004b; Ernst and Bulthoff, 2004). In addition, by contrasting two multisensory conditions we explicitly excluded any additional multisensory benefit arising from basic acoustic co-stimulation, which have been documented extensively in previous research. Visual targets could take one of 16 configurations (**Figures 1B,C**) and performance was analyzed separately at central (5°) and peripheral (14°) locations. Of the eight configurations at each eccentricity the informative sound (but not the non-informative sound) disambiguated four of these, reducing uncertainty by a factor of two (**Figures 1B,C** blue targets). These remaining four (informative sound) or eight (non-informative sound) target configurations belonged to two categories that were probed by the respective task. In the detection task this was the position relative to the horizontal midline (above or below) while in the discrimination task this was the orientation of the letter (upright or upside down). Note that by definition both tasks operate in different feature domains. The relevant feature for the detection task is spatial position while for the discrimination task it is a configural property of the letter shape. The informative sound reduced spatial uncertainty (along a task irrelevant dimension) in the detection task, while it reduced uncertainty in feature space (letter type) in the discrimination task. Importantly, for both tasks the task-relevant variable spanned one axis of the feature domain while the sound reduced uncertainty along another axis of the feature domain. The sound manipulation hence constituted a complementary (“orthogonal”) manipulation in both tasks and the design ensured that the dimensionality of stimulus domain was the same and was similarly reduced by the informative sound in both tasks. During each task we presented targets at two different intensities (brightness levels) to account for previously reported decencies of audio–visual interactions on the effectiveness of visual stimuli (Stein and Stanford, 2008; Kim et al., 2012; Otto et al., 2013).

### RESULTS FOR THE DETECTION TASK

**FIGURE 2:**
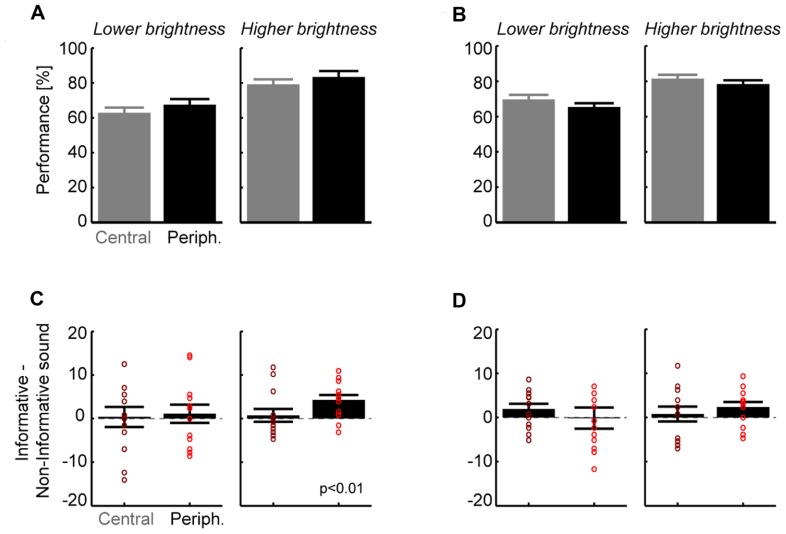
**Results for detection (A,B) and discrimination tasks (C,D).(A)** Group level performance for the detection task, separately for each target brightness and eccentricity (mean and SEM; *n* = 13). Overall performance did not differ between eccentricities but was higher for the brighter targets as expected. **(B)** Contrast between informative and non-informative sounds for the detection task. Bars denote group level results (mean and SEM), dots indicate individual subjects. Only the difference for the brighter target and peripheral locations was significant (*t* = 3.7, df = 12; *p* < 0.01; *p* < 0.05 after Bonferroni correction). **(C)** Group level performance for the discrimination task. Again performance did not differ between locations but was higher for brighter targets as expected. **(D)** Contrast between informative and non-informative sounds for stimulus discrimination. No difference reached statistical significance but there was a trend for the brighter target and peripheral locations (*t* = 2.0, df = 12, *p* = 0.06 uncorrected).

Across subjects (*n* = 13) performance levels were 65 ± 3 and 81 ± 3% correct for the lower and brighter visual intensities (**Figure 2A**; pooled over all other parameters), showing that the subject-specific selection of intensities was successful in providing low and intermediate levels of performance. Visual targets were scaled in size with retinal eccentricity and we expected no difference between eccentricities. Indeed, a separate analysis for each eccentricity revealed no significant difference between central and peripheral locations for the lower (5°:63 ± 3% vs. 14°:67 ± 3%, paired *t*-test *p* > 0.05; **Figure 2A**) or higher visual intensity (79 ± 3 vs. 83 ± 3%, *p* > 0.05). In addition, performance levels were comparable for stimuli in the upper or lower hemifields (*p* > 0.05 both intensities; e.g., 80.4 ± 3 vs. 82 ± 2% at higher intensity). This shows that behavioral performance was unbiased and balanced across spatial target positions. The main effect of interest was the difference in performance between informative and non-informative sounds (**Figure 2B**). Comparison between sound conditions revealed that for both intensities the improvement in performance (informative–non-informative sound) was larger for peripheral (lower intensity: 1.1 ± 2.0%; higher intensity 4.3 ± 1.1%) than for central targets (0.4 ± 1.9 and 0.7 ± 1.3%). Statistical analysis showed that this difference was significant at peripheral locations for the higher intensity (*t* = 3.7, df = 12; *p* < 0.01; *p* < 0.05 after Bonferroni correction), with 11 of 13 subjects (85%) showing a consistent effect. Differences for central locations or the lower intensity were not significant (*p* > 0.05). This shows that an additional sound reducing the overall spatial uncertainty about the visual target enhances detection performance at a peripheral but not at a central location.

### RESULTS FOR THE DISCRIMINATION TASK

Across subjects performance levels were 67 ± 2 and 80 ± 2% correct for the lower and brighter visual intensities (**Figure 2C**; pooled over all other parameters). In addition, performance levels were comparable to those in the detection task for each of the intensities (paired *t*-test, *p* > 0.05). Separate analysis for each eccentricity revealed no significant difference between central and peripheral locations for the lower (5°:69 ± 3% vs. 14°:65 ± 3%, paired *t*-test *p* > 0.05) or higher intensity (81 ± 2 vs. 78 ± 2%, *p* > 0.05). Performance was comparable across left and right hemifields (e.g., higher intensity: 81.2 ± 3 vs. 79.2 ± 2%, *p* > 0.05 for both).

As for the detection task we found that the differences between sound conditions were small for central locations (lower intensity: 1.9 ± 1.2%; higher intensity 0.8 ± 1.5%) but were larger for the peripheral location at the higher intensity (-0.2 ± 1.9 and 2.4 ± 1.1%). Statistical analysis showed that none of these differences was significant (*p* > 0.05) but there was a trend for the peripheral location at higher intensity (*t* = 2.0, df = 12, *p* = 0.06 uncorrected; **Figure 2D**). Discrimination performance was hence not significantly affected by the informative sound beyond any influence of basic acoustic co-stimulation provided by the non-informative sound.

## DISCUSSION

We studied the enhancement of visual detection and discrimination by auxiliary sounds. Specifically, we quantified the enhancement provided by sounds that reduce the overall uncertainty about the visual stimulus configuration beyond any enhancement provided by basic acoustic co-stimulation. This revealed a general trend for stronger enhancement at peripheral locations in both tasks, but a statistically significant effect was found only for the detection task and only at peripheral locations, despite the overall performance being similar across tasks and visual eccentricities. Our results suggest that there may be topographic differences in auditory modulation of basic visual processes and that these possibly differentially affect the detection and discrimination of visual objects. Overall this raises several interesting questions for future work.

So far, only few studies have investigated differences in audio–visual interactions with regard to visual eccentricity or task nature, and most work focused on a single task or eccentricity. Those who did consider multiple factors mostly focused on either the eccentricity or task aspect and collectively provided rather conflicting evidence. For example, a study by Fiebelkorn et al. (2011) found that the detection of near threshold stimuli was enhanced by sounds irrespective of eccentricity or the spatial alignment of sound source and visual target. While this can be taken as evidence for an overall enhancement of visual detection by acoustic inputs regardless of multisensory congruency, other studies reported more specific effects (Frassinetti et al., 2002; Bolognini et al., 2005). In addition, previous work found differential multisensory enhancement depending on the visual pathways involved. Leo et al. (2008) used color manipulations to specifically involve or avoid the koniocellular pathway and found an interaction of pathway and multisensory reaction time benefits at central and peripheral locations. The later study hence suggests at least some functional specificity with respect to target eccentricity. An eccentricity dependency of audio–visual interactions was further suggested by observations that the double-flash illusion, where sounds alter the number of perceived visual stimuli, appears more efficiently in peripheral compared to central visual locations (Shams et al., 2002). Other studies directly compared target detection and discrimination tasks at a fixed retinal position. For example, Jaekl and Harris (2009) found that uninformative sounds enhance the detection but worsen the discrimination of stimulus orientation at central locations, a finding also consistent with work on the perception of transient luminance changes (Andersen and Mamassian, 2008). In contrast to this, a recent study found that both detection and discrimination of centrally presented targets was enhanced by auxiliary sounds (Chen et al., 2011). Extending beyond this previous work, our results provide a simultaneous assessment of both detection and discrimination performance at central and peripheral retinal locations within the same subjects and using similar stimuli. Our results show that auditory influences on visual detection may be eccentricity dependent and provide support for the notion that auditory facilitation of basic visual perception can be specific with regard to perceptual function.

Eccentricity dependent acoustic influences on vision could be expected based on anatomical connectivity and functional considerations. Tracer studies revealed direct connections from auditory cortices to primary and secondary visual areas, with stronger projections from higher auditory areas (Falchier et al., 2002, 2010; Rockland and Ojima, 2003). Notably, these anatomical connections are rather weak within central representations of the visual field but are about tenfold stronger within peripheral representations, especially beyond 10° of eccentricity (Falchier et al., 2002). A direct impact of these projections on perception should hence be eccentricity dependent. Already primary auditory fields provide a high level representation of the auditory environment (Fritz et al., 2007; Nelken, 2008; Nelken and Bar-Yosef, 2008; Bizley et al., 2013) and definitely could relay information about sound localization to visual areas (Recanzone, 2000; Bizley and King, 2008). Thereby auditory cortices could directly relay the spatial information that the informative sounds provided in our detection task, predicting an eccentricity dependent enhancement of detection, just as observed. Neural activity within early auditory areas also shows sensitivity to more complex sound attributes such as speech tokens or vowels (Engineer et al., 2008; Bizley et al., 2013) but it remains unclear whether they provide sufficiently specific information to identify letters. Direct cortico-cortical projections may hence not mediate the kind of information provided by the informative sound in our discrimination task. As a result, the differential effects seen in the present study for task nature and eccentricity is compatible with the nature of the information represented in those auditory areas projecting to peripheral visual representations.

Still, while our data are consistent with a role of these direct anatomical projections in mediating the sound induced facilitation, the behavioral data cannot pinpoint the specific anatomical pathway or areas involved and the functional implications of these direct anatomical projections remain difficult to predict. Spatially resolved measurements of brain activity are required to determine whether these projections between early sensory areas directly influence perception or whether top-down projections from higher level areas are more important. Recent work in rodents has provided initial results suggesting that these direct projections indeed have an effect on neural responses within V1 and on behavior (Iurilli et al., 2012). Future work is required to directly localize the key regions mediating the multisensory perceptual benefits seen in tasks such as used here, and within the human brain. A differential effect on stimulus detection or discrimination can also be expected based on computational–perceptual mechanisms suggested to underlie low level auxiliary multisensory interactions (Lippert et al., 2007; Chen et al., 2011). Based on psychophysical studies and theoretical reasoning previous work suggested that acoustic influences on visual perception could enhance perceptual performance by reducing the temporal uncertainty about stimulus occurrence, by increasing the gain of sensory representations, or by involving the deployment of attentional resources (Chen et al., 2011). In the present study we contrasted informative and non-informative sounds, both of which reduce temporal uncertainty, hence ruling out the first potential mechanism. However, an increase in processing gain and a consequentially increased sensory energy may specifically facilitate the detection of stimuli from background, while leaving the discriminatory energy between stimuli comparable (Carrasco, 2006; Wyart et al., 2012). Similar reasoning can be applied to attentional mechanisms triggered by multisensory stimuli, and modeling work shows that feature based and spatial attention affect processing gain and tuning curves differentially (Ling et al., 2009). Both hypothesized mechanisms could hence in principle affect stimulus detection and discrimination to different degrees. While our results provide strong evidence for a differential and eccentricity dependent impact of sounds on basic visual perception, many questions regarding the perceptual mechanisms underlying an auditory facilitation of visual perception remain open. Future work, ideally based on more detailed analytical frameworks (see e.g., Otto and Mamassian, 2012; Otto et al., 2013), is required to elucidate the computational mechanisms underlying the auditory facilitation of visual perception.

We found a stronger effect of sound during the brighter visual target. At first glance this may be seen as contradicting the classical principle of inverse effectiveness (Stein and Stanford, 2008). However, it should be noted that this principle was derived based on neural responses and psychophysical studies have frequently reported that perceptual interactions can be strongest not for the weakest bur rather for intermediate stimuli. For example, the visual facilitation of speech perception is strongest at intermediate signal to noise ratios (Ross et al., 2007) and an auditory enhancement of visual motion perception was also strongest at intermediate visual intensities (Kim et al., 2012).

When interpreting the present results several points are worth noting. First, auditory stimuli were presented over headphones, which provide a less natural and more poorly localized acoustic stimulation than free field sounds. The nature of auditory presentation can have critical implications for the interpretation of audio–visual congruency effects as highlighted recently (Spence, 2013). The present tasks do not necessitate the perfect alignment of auditory and visual stimuli and merely exploit the manipulation of spatial disparity in the detection paradigm. It is hence possible that even stronger benefits of the informative sound can be observed when using free-field stimulation. However, it seems unlikely that the nature of auditory stimulation explains the absence of an effect in the discrimination task, where sounds were not spatially localized. Second, we would like to note that the sound manipulation was complementary (“orthogonal”) to the task relevant dimension in both tasks. While it was spatially orthogonal in the detection task, which by definition exploits spatial stimulus attributes, it was based on complementary feature attributes in the discrimination task (overall letter shape vs. its orientation). As such the stimuli used here should provide a comparable manipulation of audio–visual associations across both tasks. Third, we quantified multisensory benefits that were provided by the informative sound in addition to basic acoustic co-stimulation. Previous work has documented the benefits of multisensory co-stimulation in great detail (see e.g., Chen et al., 2011 for a discussion) but it remains unclear to what degree facilitatory mechanisms based on specific feature information and general alerting effects interact. Future studies may benefit from including both manipulations within a single task design. Last but not least, and to avoid misunderstandings, note that we used the term detection as referring to the differentiation of spatial stimulus positions. One can also consider this a discrimination of spatial locations, but we used the term detection here in accordance with much previous work and to provide a clearly different nomenclature. Future work could elucidate common additional differences in multisensory perception between tasks based on spatial and non-spatial attributes (Spence, 2013).

## Conflict of Interest Statement

The authors declare that the research was conducted in the absence of any commercial or financial relationships that could be construed as a potential conflict of interest.
